# Transcription Factor Map Alignment of Promoter Regions

**DOI:** 10.1371/journal.pcbi.0020049

**Published:** 2006-05-26

**Authors:** Enrique Blanco, Xavier Messeguer, Temple F Smith, Roderic Guigó

**Affiliations:** 1 Research Group in Biomedical Informatics, Institut Municipal d'Investigació Mèdica/Universitat Pompeu Fabra, Barcelona, Catalonia, Spain; 2 Grup d'Algorísmica i Genètica, Departament de Llenguatges i Sistemes Informàtics, Universitat Politècnica de Catalunya, Barcelona, Catalonia, Spain; 3 Biomolecular Engineering Research Center, Boston University, Boston, Massachusetts, United States of America; 4 Bioinformatics and Genomics Program, Centre de Regulació Genòmica, Barcelona, Catalonia, Spain; University of California San Diego, United States of America

## Abstract

We address the problem of comparing and characterizing the promoter regions of genes with similar expression patterns. This remains a challenging problem in sequence analysis, because often the promoter regions of co-expressed genes do not show discernible sequence conservation. In our approach, thus, we have not directly compared the nucleotide sequence of promoters. Instead, we have obtained predictions of transcription factor binding sites, annotated the predicted sites with the labels of the corresponding binding factors, and aligned the resulting sequences of labels—to which we refer here as transcription factor maps (TF-maps). To obtain the global pairwise alignment of two TF-maps, we have adapted an algorithm initially developed to align restriction enzyme maps. We have optimized the parameters of the algorithm in a small, but well-curated, collection of human–mouse orthologous gene pairs. Results in this dataset, as well as in an independent much larger dataset from the CISRED database, indicate that TF-map alignments are able to uncover conserved regulatory elements, which cannot be detected by the typical sequence alignments.

## Synopsis

Sequence comparisons and alignments are among the most powerful tools in research in biology. Since similar sequences play, in general, similar functions, identification of sequence conservation between two or more nucleotide or amino acid sequences is often used to infer common biological functionality. Sequence comparisons, however, have limitations; often similar functions are encoded by higher order elements which do not hold a univocal relationship to the underlying primary sequence. In consequence, similar functions are frequently encoded by diverse sequences. Promoter regions are a case in point. Often, promoter sequences of genes with similar expression patterns do not show conservation. This is because, even though their expression may be regulated by a similar arrangement of transcription factors, the binding sites for these factors may exhibit great sequence variability. To overcome this limitation, the authors obtain predictions of transcription factor binding sites on promoter sequences, and annotate the predicted sites with the labels of the corresponding transcription factors. They develop an algorithm—inspired in an early algorithm to align restriction enzyme maps—to align the resulting sequence of labels—the so-called TF-maps (transcription factor maps). They show that TF-map alignments are able to uncover conserved regulatory elements common to the promoter regions of co-regulated genes, but those regulatory elements cannot be detected by typical sequence alignments.

## Introduction

Sequence comparisons are among the most useful computational techniques in molecular biology. Sequences of characters in the four-letter nucleotide alphabet and in the 20-letter amino acid alphabet are extremely good symbolic representations of the underlying DNA and protein molecules, and encode substantial information on their structure, function, and history.

Primary sequence comparisons, however, have limitations. Although similar sequences do tend to play similar functions, the opposite is not necessarily true. Often similar functions are encoded in higher order sequence elements—such as, for instance, structural motifs in amino acid sequences—and the relation between these and the underlying primary sequence may not be univocal. As a result, similar functions are frequently encoded by diverse sequences.

Promoter regions controlling eukaryotic gene expression are a case in point. The information for the control of the initiation of the RNA synthesis by the RNA polymerase II is mostly contained in the gene promoter, a region usually 200 to 2,000 nucleotides long upstream of the transcription start site (TSS) of the gene. Transcription factors (TFs) interact in these regions with sequence-specific elements or motifs (the TF binding sites (TFBSs)). TFBSs are typically 5–8 nucleotides long, and one promoter region usually contains many of them to harbor different TFs [1]. The interplay between these factors is not well understood, but the motifs appear to be arranged in specific configurations that confer on each gene an individualized spatial and temporal transcription program [1]. It is assumed, in consequence, that genes exhibiting similar expression patterns would also share similar configurations of TFs in their promoter.

However, TFBSs associated to the same TF are known to tolerate sequence substitutions without losing functionality, and are often not conserved. Consequently, promoter regions of genes with similar expression patterns may not show sequence similarity, even though they may be regulated by similar configurations of TFs. For instance, only about 30% to 40% of the promoter regions are conserved between human and chicken orthologous genes [2], and the conservation of human–mouse orthologous promoter regions is only slightly higher than that observed in intergenic regions [3]. Indeed, despite the recent progress due to the development of techniques based on so-called phylogenetic footprinting [4], lack of nucleotide sequence conservation between functionally related promoter regions may partially explain the still limited success of current available computational methods for promoter characterization (see [5] and [6] for further information).

In the approach described here, we attempt to overcome this limitation by abstracting the nucleotide sequence, and representing a promoter region by a sequence in a new alphabet in which the different symbols denote different TFs. Using an external mapping function, for instance, a look-up table or a collection of position weight matrices (PWMs) that associates each TF to the nucleotide sequence motifs the factor is known to bind, we can translate the nucleotide sequence of the promoter into a sequence in this new alphabet. These sequences can be aligned. If the scoring of the alignment takes into account not only the presence/absence of a given symbol, but its relative position on the primary nucleotide sequence, the optimal alignment between the promoter regions of two genes with similar expression patterns may reflect the underlying common configuration of TFBSs. We refer to these alignments either as meta-alignments, as they are performed between sequences in a meta-alphabet, or map alignments, since they are obtained after mapping the nucleotide sequence in a higher order alphabet.

In this paper, first we state formally the problem of mapping a nucleotide sequence into a sequence in an alphabet of TFs denoting symbols (a transcription factor map, or TF-map), and introduce the notion of pairwise alignment between two such sequences or maps. Then, based on an early algorithm to align restriction enzyme maps [7], we develop an efficient algorithm to obtain the optimal global alignment between two TF-maps. We estimate the optimal parameters of the alignment in a set of well-characterized human–rodent orthologous gene promoters, using a number of different mapping functions (collections of PWMs representing TFBSs). Finally, we evaluate the ability to distinguish co-regulated from non co-regulated genes based on the comparison of promoter sequences. Our results, obtained in CISRED [8], a large database of human co-regulated genes, and relying on the JASPAR collection [9] to derive the TF-maps, indicate that comparisons of TF-maps are able to reveal relationships between co-regulated genes that are not detected by nucleotide sequence comparisons.

## Results

### The Algorithm

#### Translation of the promoter sequence into a TF-map.

**Figure 1:**
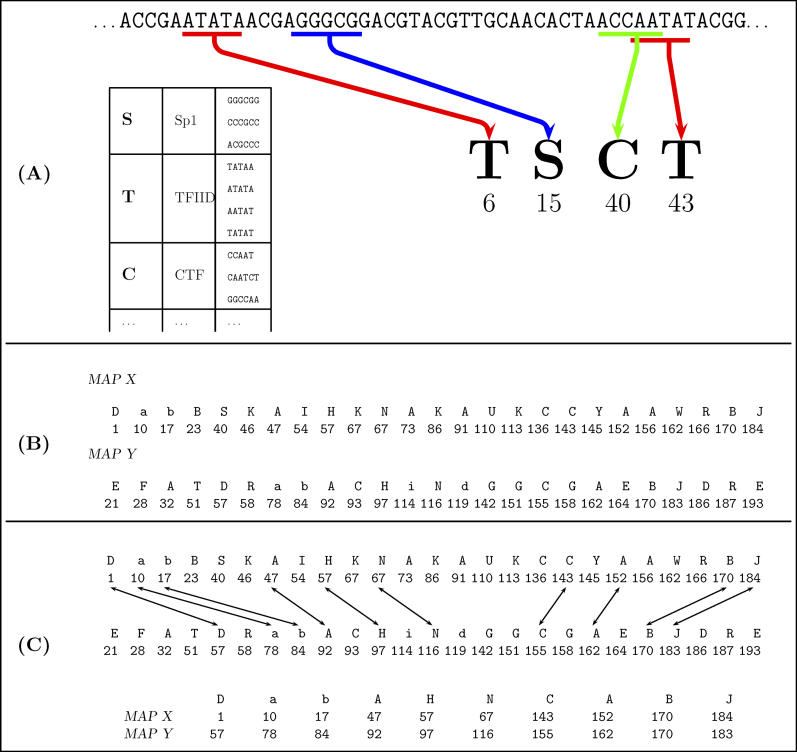
TF-Map Alignment of the Promoters of Two Hypothetical Co-Regulated Genes (A) The sequence of a promoter is searched for occurrences of known binding motifs for TFs. Matches are annotated with the position of the match in the primary sequence, and the label of the TF. Because TFs can bind to motifs showing no sequence conservation, labels of the same TF at different positions may correspond to different underlying nucleotide sequences. We refer here to these sequences of pairs (“label,” “position”) as TF-maps. TF-maps are actually more complicated. First, we do not only register the position of each match, but also its length. Second, while in the example here, sequence motifs are associated to TFs by means of a (binary) look-up table, in our work we have instead used collections of PWMs. Matches to TFBSs are thus scored, and this score is also registered. (B) TF-map of the promoter region of two hypothetically co-regulated genes *X* and *Y*. Each letter corresponds to a different TF. We assume that 200 nucleotides upstream of the annotated TSS have been considered, with position 1 corresponding to position −200 from the TSS. (C) Global pairwise alignment of the two co-regulated genes *X* and *Y*. Only positions with identical labels can be aligned. Essentially, the alignment finds the longest common substring constrained to maximizing the sum of the scores (unpublished data) of the aligned positions, and minimizing the differences in the distances on the primary sequence between adjacent aligned positions.

In our approach, we translate the nucleotide sequence of a promoter region *S* = *s*_1_ *s*_2_ … *s*_k_ into a sequence of 4-tuples *A* = *a*_1_ … *a_n_* where each 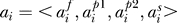 denotes the match with score 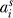 of a binding site for the TF 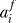 occurring between the position 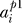 and the position 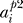 over the sequence *S*. In the work described in this paper, we obtain the translation from *S* to *A* by running on *S* a collection of PWMs representing binding motifs for TFs (such as, for instance, the collection in TRANSFAC [10]). For each match over a given threshold, we register in *A* the positions 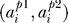 , the score 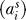 , and the label 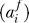 of the TF associated to the PWM. The translation preserves the order of *S* in *A*, that is if *i < j* in *A* then 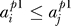 (the ≤ is because matches to different TFs may occur at the same position). We will refer to the resulting sequence *A* as a TF-map or simply a map (see Figure 1). Note that other mapping functions, instead of collections of PWMs, can also be used to translate *S* into *A*.

In the implementation here, matches to PWMs are considered strandless, that is, they are annotated at a given location, irrespective of the orientation in which they occur. While biological evidence suggests that some TFBSs are functional only when present in a given strand, in other cases TF activity appears to be independent of the orientation of the binding site [11]. Since in general we do not have information about the strand in which a binding site may be functional, we have not considered strand in our analysis.

#### Alignment of TF-maps.

The alignment of the maps *A* = *a*_1_ … *a_m_* and *B* = *b*_1_ … *b_n_* is a correspondence *T,* maybe empty, between *A* and *B* such that: 1) 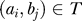 if and only if 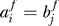 (that is, two elements are aligned if and only if they correspond to the same TF); 2) if 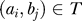 *,* then there are no other elements *b_l_* (*l* ≠ *j*) in *B* such that 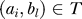 *,* nor elements *a_k_* (*k* ≠ *i*) in *A* such that 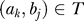 (that is, each element in *A* is aligned at most to one element in *B*, and vice versa); 3) if 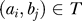 and 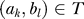 and *i* < *k,* then *j* < *l* (that is, the alignment maintains the colinearity between the sequences *A* and *B*); 4) if 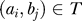 and 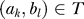 with *i* < *k* and *j* < *l,* then 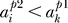 and 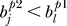 (that is, no overlap in the primary sequences is permitted between the sites corresponding to the aligned elements). Usually there are many possible alignments between two given *A* and *B* maps (see Figure 1 for an example). Given an alignment *T*

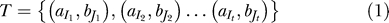
where 
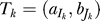
 is the match between the 4-tuple in position *I_k_* from *A* and the 4-tuple in position *J_k_* from *B,* we compute the score of the alignment *s(T)* in the following way:

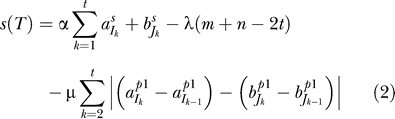



where *α, λ, μ >* 0*.* That is, the score of the alignment increases with the score of the aligned elements (*α*), and decreases with the number of unaligned elements (*λ*), and with the difference in the distance between adjacent aligned elements (*μ*).

#### Finding the optimal alignment.

The optimal alignment between two given maps *A* and *B* is the one scoring the maximum among all possible alignments. To obtain such an alignment efficiently, we have implemented an algorithm reminiscent of that proposed by Waterman et al. [7] to align and compare restriction enzyme maps. This algorithm was developed to find the distance between two homologous restriction maps in terms of minimum weighted sum of genetic events necessary to convert one restriction map into another, where the genetic events are the appearance/disappearance of restriction sites and changes in the number of bases between restriction sites. A restriction map was defined as a sequence of pairs *A* = *a*_1_ … *a_n_* where each pair 
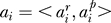
 denotes the restriction endonuclease 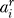 occurring at position 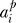 over a nucleotide sequence *S*. Then, the minimum distance *D_ij_* required to convert the map *A* = *a*_1_ … *a_i_* into the homologous map *B* = *b*_1_ … *b_j_,* where the site 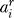 is equal to the site 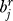 *,* was obtained by applying the following recursion:

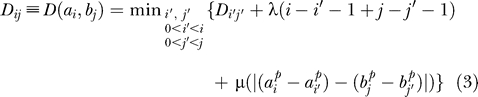
where λ is the penalty associated with the appearance/disappearance of sites, and *μ* is the penalty associated with the difference in distance between consecutive aligned sites. Here, to align TF-maps *A* and *B*, we adapted the recursion in [7] to optimize similarity instead. In addition, we included a term (*α*) into the scoring function to weight the scores of the TFBSs. We also explicitly prohibited overlap between the sites. Thus, the maximum similarity between TF-maps *A* = *a*_1_ … *a_i_* and *B* = *b*_1_ … *b_j_,* where the site 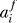 is equal to the site 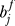 *,* can be computed as:

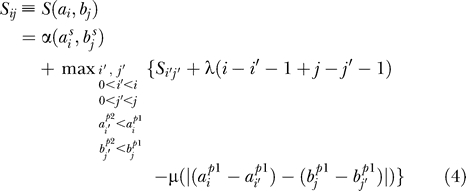



#### Naive implementation.

A naive implementation of the recursion above (Equation 4 ) involves the recursive filling of the cells *S_ij_* in the matrix *S* [7]. In the pseudocode below (Algorithm 1), the elements of the maps *A* and *B* are represented as structures *a_i_* and *b_j_*, with the functions *factor, score, pos1,* and *pos2* returning the values of the corresponding fields. The variable *currentSim* stores the optimal score so far computed. The resulting meta-alignment can be easily retrieved using a supplementary structure *path(i,j)* which points to the previous cell in the optimal path leading to cell *S_ij_*. In addition, for each cell *S_ij_,* the function *ComputeInitialSimilarity* calculates the initial score of a hypothetical alignment that includes only *a_i_* and *b_j_.* Note that to compute the optimal score at *S_ij_* with this algorithm, all the cells *S_kl_* (*k* < *i*,*l* < *j*) need to be explored. Therefore, if the lengths of the TF-maps *A* and *B* are *m* and *n,* respectively, the cost of computing *S*(*A*,*B*) = *S*(*a_m_*,*b_n_*) is *O*(*mn · mn*) = *O*(*m* ^2^ *n* ^2^). Under the assumption that *m* and *n* are similar lengths, the final cost function is *O*(*n* ^4^).

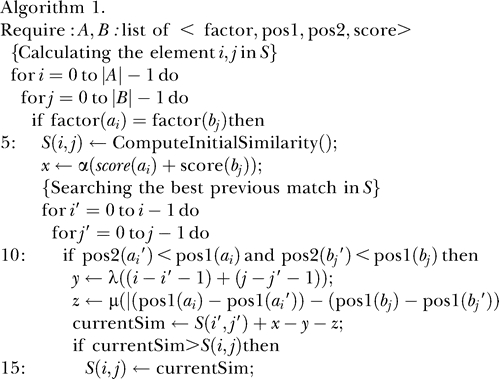



#### Enhanced implementation.

Myers and Huang [12] described an improved algorithm for computing in *O*(*mn*(log *m* + log *n*)) time the minimum distance between two restriction maps of length *m* and *n,* respectively, under the original framework proposed by Waterman et al. [7]. The algorithm is basically a sparse dynamic programming computation in which candidate lists are used to model the future contribution of all previously computed cells in distance matrix *D* to those yet to be computed. The cells in the list that cannot affect the values of any cell to be computed are eliminated from the list. The key concept of this algorithm is the mapping of the original matrix *D* to another matrix in which each cell is indexed by the positions of the sites in the original sequences, and not by their positions in the maps. During the computation, this matrix is partitioned into intervals for which only a representative cell is used to compute the best alignment ending at each match in a given interval. Here, we cannot directly export this strategy, because in contrast to the restriction enzyme maps which are points in the sequence, TFBSs are sequence intervals (having, thus, two dimensions). In addition, different TFBSs can start at the same point, but end at different positions. Because we explicitly prohibit overlapping between TFBSs in the alignments, the assignation of a cell representative within a given interval must not be irreversible. However, we have still taken advantage of the extreme sparsity of the matrix *S* when aligning TF-maps.

**Figure 2:**
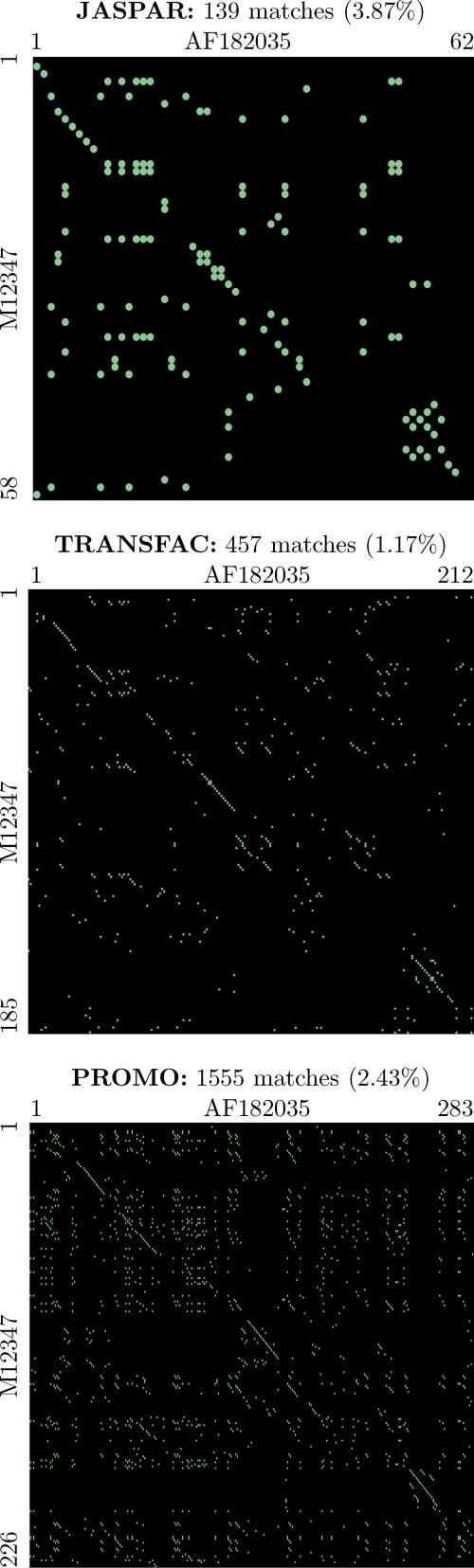
Graphical Representation of the Sparse Dynamic Programming Matrix *S* When Obtaining the TF-Map Alignment between the Human and Mouse Promoters of the *skeletal alpha-actin* Gene, Using Different Collections of PWMs for TFBSs The axes of the matrix list the TF labels of the predicted TFBSs in the human and mouse promoters. Despite the differences in the total number of predicted TFBSs depending on the collection, the occupancy of the matrix remains consistently low.

Note that, in general, the probability of matching two elements from two sequences of characters that follow a uniform random distribution is inversely proportional to the size of the character alphabet. For instance, the probability of matching two nucleotides when comparing two random DNA sequences in the four-letter alphabet is about 0.25. In an alphabet of about 100 characters—the order of magnitude of the alphabets of symbols denoting TFs that we are considering here—such a probability would be about 0.01. When aligning sequences in alphabets of such sizes, the matrix *S* above, which only takes values for match positions between *A* and *B,* becomes therefore extremely sparse. Indeed, Figure 2 displays the occupancy of the matrix *S* corresponding to the alignments of the TF-maps obtained on the human and mouse promoters of the *skeletal muscle α-actin* gene.

We have used three different collections of PWMs for TFBSs (see Materials and Methods) to obtain the TF-maps of both promoter sequences. In all cases, despite the differences in the lengths of the obtained maps, the occupancy of the matrix *S* is well under 5%. In the algorithm below (Algorithm 2), we substitute the two internal nested loops by a list *L* to register the coordinates of the match cells in the sparse matrix *S*. Each node of *L* is represented as structures *p* and *n* with the functions *abscissa* and *ordinate* returning the corresponding coordinates. Thus, to compute the optimal score at the cell *S_ij_,* only the non-empty cells in *S* need to be accessed. In addition, we maintain the list sorted by optimal score, so that the cell scoring the maximum value is at the beginning of the list. Scanning the list from the beginning to the end implies that, in most cases, only a few nodes will need to be accessed before a critical node is reached beyond which the optimal score cannot be improved.

While investigating the exact complexity of this algorithm is difficult—depending mostly on the size of the input maps and the sparsity of the resulting matrix *S—*the expected time cost analysis can be performed. The *O*(*n* ^4^) cost of the naive algorithm can be explained in terms of (a) a first quadratic term derived from the obligatory comparison between all of the TFBSs of both maps to detect the match cells and (b) a second quadratic term necessary to search for each match the best adjacent previous pair in the optimal TF-map alignment. In this enhanced algorithm, the contribution (a) is inevitable so that the lower bound of the cost function is the number of matches between both TF-maps, that is *O*(*n* ^2^). However, the substitution of the two inner loops for a list of cell matches sorted by optimal score does affect the contribution (b). Thus, such a term is now equivalent to the expected number of consulted elements of the ordered list *L* to compute each *S_ij_* value. This expectation can be approximated to

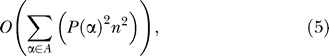
where *A* is the set of symbols (in our case the alphabet of TFs) and *P*(*α*) is the probability to match the symbol *α* in a random trial (it is a particular case of the sequence comparison by hashing, see Theorem 8.1 in [13]). Therefore, under the previous hypothesis of a comparison between two TF-maps in an alphabet of 100 characters that follows a uniform random distribution (*P*(*α*) = 0.01, only 1% of the matrix is occupied), the expected value of the contribution (b) is *O*(0.01*n* ^2^).

The empirical results obtained during the program training confirmed such analysis. On average, on the order of 200 million elements were consulted by the naive algorithm during the optimization. In contrast, the enhanced algorithm only needed to access nearly two million elements to compute the same set of alignments (see Figure S1).


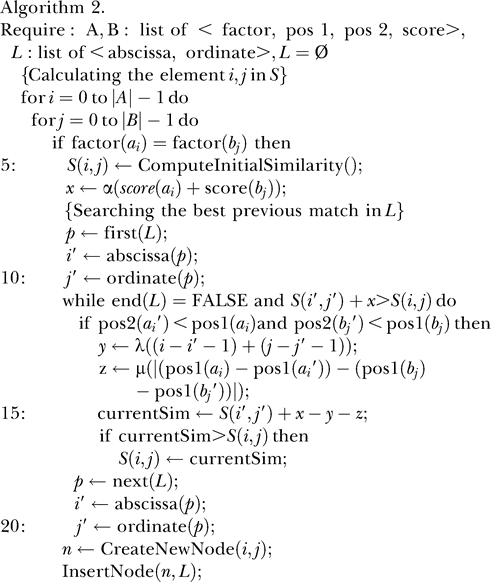


### Using TF-Map Alignments to Characterize Promoter Regions of Co-Regulated Genes in the Absence of Sequence Similarity

Training results (see Materials and Methods) indicate that alignments of TF-maps can contribute—together with other tools, such as primary sequence alignments—to the characterization of the promoter region of co-regulated genes. This contribution is mostly obtained through the substantial reduction of the overwhelming number of candidate TFBSs that PWMs and other pattern-based searches typically produce. The co-regulated genes in the test case of the training (see Materials and Methods), however, were orthologous human–mouse pairs. The promoter regions of such pairs show substantial sequence conservation [3]. It can be argued that under such circumstances map alignments may not be much more informative than primary sequence alignments. Note that, in general, good alignments at the primary sequence level will inevitably result—given the low specificity of the PWM search—in good map alignments, although such map alignments may bear little relationship to the underlying conserved configurations of TFBSs.

To assess to what extent good TF-map alignments are simply a reflection of underlying sequence conservation, we have compared the meta-alignments obtained using the JASPAR*_TOP_*_50_ collection of matrices (see Materials and Methods) in the 200 nucleotides of the promoter region of the 36 gene pairs from the HR set that we used in the training (see Materials and Methods), with the meta-alignments obtained in fragments of 200 nucleotides from intergenic (2,000 nucleotides upstream of the TSS), 5′UTR (downstream of the TSS), coding (downstream of the translation start site and considering only coding DNA), intronic (downstream of the first intron junction), and downstream (downstream of the transcription termination site) sequences. We have computed the average score of the map alignments in each of the genomic regions and have identified, for each homologous pair, the genome regions in which the alignment produces the highest score. We have performed the same exercise using global pairwise sequence alignments (obtained with CLUSTALW, [14]). Results appear in Table 1 (top). As expected, nucleotide sequence alignments score the highest in the coding regions (in 26 out of 36 cases), followed by the alignments in the promoter (five out of 36) and 5′UTR regions (four out of 36). The scores of the sequence alignments show that promoter regions are less conserved than coding regions, and have a level of conservation similar to that observed in 5′UTRs. Despite this, TF-map alignments score the highest in the promoter regions (in 25 out of 36), where the average score of map alignments is almost twice as high as that of the coding regions. Only in six out of 36 cases does the TF-map alignment score the highest in coding regions. Interestingly, while intron sequences in the orthologous human–mouse pairs are much less conserved than 5′UTRs, TF-map alignments have a similar score in both regions. In fact, in three cases, TF-map alignments have the highest score in first introns, while only in one case in 5′UTRs. This is consistent with the fact that first introns are known to often contain regulatory motifs.

**Table 1:**
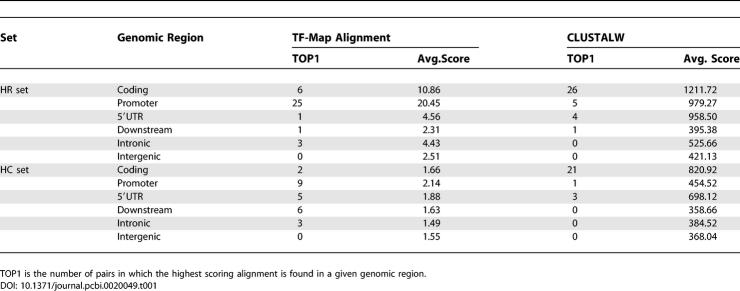
Sequence and TF-Map Alignments of Different Genomic Regions between the Human and Mouse Orthologous Pairs in the HR Set and between the Human and Chicken Orthologous Pairs in the HC Set

To measure the ability of TF-map alignments to detect conserved regulatory elements at larger evolutionary distances—at which the degree of sequence conservation may be negligible—we have carried out the same analysis on a set of human–chicken orthologous pairs derived from the HR set. Using the RefSeq gene set as mapped into the UCSC genome browser, we have identified the chicken ortholog for 25 genes in the HR set. We refer to the resulting set of human–chicken gene pairs as the HC set. As before, we have compared promoter, intergenic, 5′UTR, coding, intronic, and downstream sequences between the orthologous human–chicken genes using both TF-map alignments based on JASPAR*_TOP_*_50_ and sequence alignments using CLUSTALW. Results appear in Table 1 (bottom). While, as expected, the scores of the alignments are, in both cases, clearly lower for human–chicken than for human–mouse comparisons, the same relative trends can be observed, with sequence alignments being most significant between coding regions, and TF-map alignments between promoter regions. However, while coding sequences are still distinctively conserved between human and chicken, similarity in promoter sequences degrades substantially. Indeed, in contrast to human–rodent comparisons, 5′UTRs are, for instance, clearly more conserved than the promoters between human and chicken orthologous genes. Despite this lack of sequence similarity in the human–chicken promoter pairs and the fact that we trained our algorithm specifically on human and rodent genes, the TF-maps remarkably still score the highest in these regions (in nine out of 25). Interestingly, TF-map alignments are able to score comparatively high in downstream regions even though they do not appear to exhibit sequence conservation; regulatory motifs have been occasionally reported on these regions. Overall, these results indicate that alignments of TF-maps are able to detect conservation of regulatory signals, which cannot be detected by sequence similarity alone.

#### Detection of co-expression by promoter comparisons in the CISRED database.

We expect, therefore, the map alignments to be particularly useful to characterize promoter regions of co-regulated genes in the absence of sequence conservation. In such cases, the map alignments can help to recover conserved configurations of TFBSs that primary sequence comparisons would not. It is important to stress in this regard that the match state in the alignment of TF-maps is defined based on the TF label, and not based on the label of the specific binding site. Because a given TF can be associated to different binding sites (for instance, the approximately 90 TFBSs in the HR set correspond only to about 30 TFs), an alignment of TF-maps can include the alignment of TFBSs that show no sequence conservation.

Many examples could be found in which map alignments produce a better characterization of the promoter region of co-regulated genes than that obtained through primary sequence alignments. We would like, however, to move beyond such anecdotal evidence, and have a more exhaustive evaluation of the power of TF-map alignments to characterize promoter regions of co-regulated genes in the absence of sequence similarity. Toward such a goal, we have used the set of co-regulated genes in the CISRED database [15]. The CISRED database is primarily a collection of conserved regulatory sequence elements identified by a genome-scale computational system that uses pattern discovery, similarity, clustering, co-occurrence and co-expression calculations. CISRED includes, as well, a database of high-confidence co-expressed gene pairs [8], obtained from cDNA microarray hybridization, SAGE, and other experiments, as well as from Gene Ontology (GO, [16]) analysis. Version 1 of CISRED high confidence co-expression human set contains 60,912 co-expression gene pairs for 5,562 genes. Because of the criteria to establish co-regulation within CISRED, we do not expect strong bias toward co-expression pairs sharing strong sequence similarity in their promoter regions.

We thus performed the following experiment: we compared the promoter region of each gene *x* in the CISRED set with the promoter regions of the genes co-regulated with *x*, *coreg*(*x*), and with the promoter region of the genes not co-regulated with *x*, 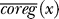 . Even though the promoter of the gene *x* may not show stronger sequence similarity with the promoters of the genes in *coreg*(*x*) than with the promoters of the genes in 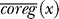 , our assumption is that it will still share some common regulatory signal (maybe very weak) with the promoters of (at least a fraction of) the genes in *coreg*(*x*), whereas no common signal will be shared between the promoter of *x* and the promoters of the genes in 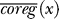 . Our hypothesis is therefore that alignments of TF-maps will be superior in detecting such signals to alignments of the primary nucleotide sequence. We proceeded in the following way: we used ENSMART [17] to extract 500 nucleotides upstream of each gene in CISRED according to genome coordinates in ENSEMBL. We used 500 nucleotides upstream here, instead of 200 nucleotides as before, because of the intrinsic imprecision of ENSEMBL when annotating the coordinates of the TSS. We obtained such a sequence for 5,333 out of 5,562 CISRED genes and considered it the promoter region of the gene. For this set of 5,333 genes, 56,632 co-expression gene pairs are described in CISRED. We next used the collection of matrices in JASPAR*_TOP_*_50_ (see Materials and Methods) to obtain the TF-maps of each promoter region. Then for each gene *x* we obtained the optimal map alignment with each gene in *coreg*(*x*) and in 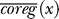 . We used the enhanced algorithm described earlier, with the optimal parameters estimated in the Material and Methods section. Finally, we determined whether the scores of the map alignments between the promoter of gene *x* and the promoters of the genes in *coreg*(*x*) were significantly higher than the scores of the map alignments between the promoter of gene *x* and the promoters of the genes in 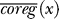 . Because the scores of the optimal TF-maps alignments follow, as optimal sequence alignments, a Gumbel or extreme-value distribution (see Figure S2 and Figure S3), we calculated the Wilcoxon test to assess this hypothesis. We obtained 42,756 non-void *coreg*(*x*) alignments and 20,600,640 non-void 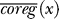 alignments. 4,784 genes in CISRED had non-void alignments for both the *coreg*(*x*) and the 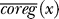 sets. The average score of the *coreg*(*x*) alignments was 6.02, and the average length was 2.13 sites. For the 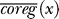 alignments, the values were 5.57 and 2.06, respectively. For 97 genes, the score of the *coreg*(*x*) alignments was significantly higher than that of the 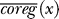 alignments at a significance level of *p* = 0.01. At a *p*-value of 0.001, the number was 23. Because CISRED is partially based on microarray experiments, one could argue that cross-hybridization with recently duplicated genes may artefactually bias these results. However, no duplicated copies of genes exist in the sets of co-regulated genes with the 97 positive cases above (see Dataset S1).

We performed the same experiment, using BLASTN [18] instead to compare the promoter region of each gene *x* in the CISRED set with the promoters of the genes in *coreg*(*x*) and 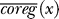 . BLASTN was used with the parameters word size 7 and expectation value 10 so that short stretches of conservation could also be retrieved. In each comparison, we identified the score of the best HSP. We obtained 981 *coreg*(*x*) alignments and 445,371 non-void 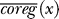 alignments. 653 genes in CISRED had BLASTN alignments in both the *coreg*(*x*) and the 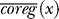 sets. The average score of the *coreg*(*x*) alignments was 29.9, and the average length was 51 nucleotides. For the 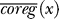 alignments, the values were 24.3 and 40.5, respectively. For 11 genes, the score of the *coreg*(*x*) alignments was significantly higher than that of the 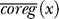 alignments at a significance level of *p* = 0.01; there was only one gene for which the score of the *coreg*(*x*) alignments was significantly higher than that of the 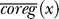 alignments, at a significance level of *p* = 0.001.

We have investigated whether differences in conservation of regulatory elements could be found between promoters associated to CpG islands (CpG+) and promoters not associated to them (CpG−). CpG− promoters have been linked to tissue-specific expression patterns [19,20], and therefore they could be overrepresented in the set of co-expressed genes for which we have been able to identify conserved regulatory motifs. We computed for each gene the GC content and the CpG score as defined by [19]. The presence of a CpG island on a window (−100:+100) centered around the TSS of a gene is accepted when its GC content is greater than 0.5 and when its CpG score is greater than 0.6 (CpG+); otherwise it is classified as a CpG negative gene (CpG−). Genes lacking CpG islands around their TSS have been shown to have a more tissue-specific expression pattern [19]. Based on these considerations, 3,844 out of the 5,333 promoters (72%) were identified as CpG+ genes, while only 1,489 (28%) were classified as CpG−. Among the 97 genes for which the score of the *coreg*(*x*) TF-map alignments was significantly higher than that of the 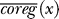 alignments at a significance level of *p* = 0.01, 63 were CpG+ (65%). At a *p*-value of 0.001, the number of CpG+ genes was 13, out of a total of 23 (56%). It, thus, indeed appears that genes with CpG− promoters are slightly overrepresented in the set of co-regulated genes with conserved (specific) regulatory signals.

As it is possible to see, despite the general poor ability of both the sequence alignments and the TF-maps to uncover relationships between the promoters of the co-regulated genes in CISRED, it is clear that TF-map alignments are able to detect more relationships than BLASTN alignments (97 versus 11 at a *p*-value < 0.01, 23 versus one at a *p*-value < 0.001). It can be argued that this is partially an artefact, resulting from BLASTN reporting only sequence alignments over a given threshold, while non-void TF-map alignments are always produced, provided that the maps to align share at least one common element. In fact, given the number of genes for which valid alignments are obtained, at a *p*-value < 0.01 there are twice as many cases in which *coreg*(*x*) scores are significantly higher than 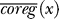 as expected if there was actually no difference in the distributions of scores, both using TF-map and sequence alignments. At a *p*-value < 0.001, however, the number of cases in which *coreg*(*x*) scores are significantly higher than 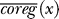 coincides with the expected value using BLASTN, but it is five times the expected value using TF-maps. We believe that this indicates that, even after taking into account the effect of the different number of total alignments reported, the TF-map alignment algorithm is superior to BLASTN in detecting relationships between the promoter regions of co-regulated genes. Indeed, among the 445,371 total BLASTN alignments obtained, there are 981 alignments between co-regulated genes, while the 445,371 top scoring TF-map alignments obtained include 1,240 alignments between co-regulated genes. Interestingly, there are only 148 alignments in common between both approaches, indicating that they could be used to complement each other.

It could be argued that the superiority of the TF-map over sequence alignments has little to do with the alignments and more to do with the maps. In other words, we would have obtained similar results if we were to simply score the proportion of TF labels common to the compared promoter regions—without the need for an alignment. Therefore, we have computed such a score for each pair of genes in CISRED: if *p* and *q* are the sets of elements in the TF-maps of the promoters to be compared, we have computed | *p* ∩ *q* |^2^ / | *p* | · | *q* |, where *|p|* is the size (cardinality) of the set *p*. Among the 445,371 top-scoring comparisons, 1,072 corresponded to co-regulated genes (with only 394 gene comparisons in common with the TF-map alignment approach), a value intermediate between that obtained with sequence and with TF-map alignments. This reflects that conservation of the relative position of the TFs along the primary sequence, and not only common presence, is indicative of gene co-regulation. Conservation of relative position can only be captured by TF-map alignments.

As an example, Table 2 summarizes the TF-map alignments obtained when aligning the promoter region of the *transthyretin* gene (TTR), with each one of its co-regulated genes in CISRED. TTR is a serum carrier protein expressed in liver and brain. The regulatory regions that control the TTR expression in liver have been experimentally determined [21] and consist of a 100-nucleotide enhancer located at −2,000 nucleotides upstream of the TSS and a proximal promoter region between −200 and −90 nucleotides upstream of the TSS (relative to the coordinates in the ENSEMBL entry). This proximal region comprises six binding sites (coordinates relative to TSS of the *transthyretin* gene as in the ENSEMBL database): HNF-1 (−137,−109), HNF-3 (−140,−128 and −106,−91), HNF-4 (−151,−140), C/EBP binding (−195,−177 and −135,−112). The TATA box is located at −30. CISRED lists 105 genes co-regulated with TTR. Interestingly, while BLASTN is unable to detect any sequence similarity between the promoter of TTR and that of its co-regulated genes, TF-map alignments are obtained in 83 cases, and scored significantly (*p*-value < 0.001). We have reconstructed the structure of the TTR promoter from the elements that appear in the TF-map alignments. A total of 35 TFBSs were initially mapped with JASPAR*_TOP_*_50_ in the TTR promoter. For each predicted TF, Table 2 lists the number of TF-map alignments between TTR, and its co-regulated genes in which the TF appears. Only elements appearing in at least five alignments are reported. No matrices for the detection of C/EBP and HNF-4 were included in the JASPAR*_TOP_*_50_ collection that was used to perform the test. However, the meta-alignments were overrepresented in the other experimentally annotated sites, HNF-1, HNF-3, and TATA, exactly in the region where promoter activity has been reported. The binding of HNF-3 to the site −140,−128 is not directly reported. The TF-map alignments, however, are highly enriched in the HFH-3 factor (HNF3/fork head homolog, [22]) at this region. In fact, both share a similar consensus-binding sequence in TRANSFAC [10]: TRTTTRTTT for HFH-3 and TRTTTRYTT for HNF-3.

**Table 2:**
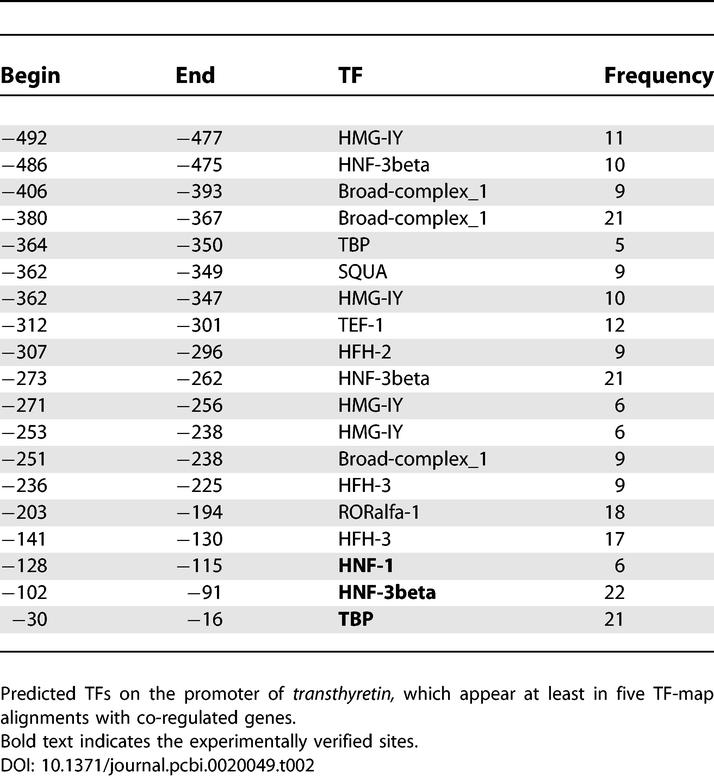
Summary of the TF-Map Alignments Obtained between the Promoter of the *transthyretin* Gene and the Promoters of the Genes Co-Regulated with It according to the CISRED Database

## Discussion

Much of the biology of the past decades has been based on the technological advances that have accelerated our ability to sequence DNA and proteins. It is certainly in the sequence of the genome that the biological traits of organisms are encoded. While we have a relatively good understanding of some of the basic mechanisms involved in the processing of the information encoded in the DNA sequence, it is in general very difficult to predict the biological traits—even at the molecular level—from the nucleotide sequence alone. Gene promoters are a case in point: while the sequence of the promoter is likely to contain most of the information to control the expression of a gene, it is currently impossible to predict the expression pattern of a gene from the analysis of its promoter sequence alone.

While inferring function directly from sequence is thus far from trivial, it is still true that because sequence encodes function, similar sequences often encode similar functions. Sequence comparisons, therefore, are an extraordinary tool to infer functional relationships: through sequence comparisons the function of known sequences can be extrapolated to newly obtained ones, and the specific sequence motifs responsible for the common functionality of a set of sequences can be identified. But sequence comparisons have limitations: often similar functions are encoded by diverse sequences. Again, gene promoters are a case in point: many TFs bind to sequence motifs which do not show sequence conservation. Thus, while through phylogenetic footprinting, conserved regulatory motifs have been on occasion uncovered in the promoters of orthologous genes [23,24], searching for common patterns through the comparison of promoter sequences in sets of co-regulated genes—as, for instance, those resulting from microarray experiments—is usually a frustrating exercise.

Here, we have attempted to address this limitation implicit in sequence comparisons, by annotating the primary sequence with predicted functional domains, and comparing the resulting annotations instead of the underlying primary sequence. If functional domains are encoded by diverse sequences, the comparison and alignment of the annotation may be more revealing of the functional relationships between sequences and of the specific domains involved in the common functionality than the comparison and alignment of the primary sequence. In particular, we have attempted this strategy for the comparison and characterization of promoter regions from genes with similar expression patterns. We have annotated the sequence with predictions of TFBSs—using a variety of popular tools and databases—and identified the predicted sites with the labels of the corresponding TFs. We have then compared and aligned the resulting sequence of labels. Because TFs can bind to sites that show no sequence conservation, their labels can be aligned which correspond to domains that, while exhibiting similar functions, may not show sequence conservation.

Precedents of this approach can be found in the literature. Quandt et al. [25], for instance, distinguish explicitly between first-level analysis of promoters, in which the nucleotide sequence is directly interrogated for the presence of regulatory motifs, and second-level methods, in which basic higher order patterns can be defined from a number of correlated first-level units. This approach is further developed in [26] and [27], where more complex composite patterns are derived capturing the functional organization of individual regulatory elements, and are then used to identify and characterize related promoter regions in absence of sequence conservation. In a related approach, Solovyev and Shamuradov [28], for instance, also use higher order information to characterize orthologous promoters. Specifically, they use linear discriminant analysis to combine a number of conservation features and nucleotides sequences of promoter regions in pairs of orthologous genes.

Here, we go one step further, and infer automatically the composite patterns by explicitly aligning the sequences of labels corresponding to TFs for which binding sites have been predicted in the compared promoters (the second-level annotation). To align these sequences of labels—to which we refer as TF-maps—we have stated the problem as a restriction enzyme map alignment, and adapted a dynamic programming algorithm developed by Waterman et al. [7]. This algorithm, as well as ours, belong to a larger class of map alignments algorithms (see also [12,29–31]). In typical alignments, the sequences are of labels denoting either nucleotides or amino acids. In map alignments, the sequences are of pairs (label,integer), where the label denotes a predicted domain or site (possibly exhibiting some behavior or functionality), and the integer the position on the primary sequence where the domain or the site has been predicted. In global pairwise sequence alignments, the goal is to obtain the alignment that maximizes the sum of the scores of the aligned positions—given the score of the individual alignments of all possible pairs of labels. In contrast, in map alignments, only positions with identical labels can be aligned and the goal is to obtain the largest common subsequence constrained to minimize the differences in distances on the primary sequence between consecutive aligned positions. Sequence and map alignments can be generalized to a broader class of alignments that includes both.

Map alignments have been mostly used to align restriction enzyme maps. In this case, the label denotes a restriction enzyme, and the integer the position on the primary sequence of the site recognized by the enzyme. Waterman et al. [7] first established the concept of map alignment and provided an algorithm for computing the optimal alignment of two maps. Later Myers and Huang [12] described an improved algorithm to efficiently find map alignments that relies on the extreme sparsity of the dynamic programming matrix in [7]—the result of the match state being defined only between identical labels. Miller et al. [29,30] introduced new algorithms that permitted the efficient search of a long map for the best matches to a shorter probe map. Huang and Waterman [31] generalized these algorithms to deal with different map errors.

In our case, the label denotes a TF, and the integer the initial position on the primary sequence where a binding motif for the TF has been predicted. There are, however, two important differences between restriction enzyme maps and TF-maps. First, while prediction of restriction sites is deterministic, producing a binary output (“site,” “no site”), prediction of TFBSs is often probabilistic and predicted sites may have an associated score. The score can usually be related to the strength of the binding of the TF to the site [32]. Because it makes sense, therefore, to prefer in TF-map alignments higher scoring sites, the score of the TFBSs needs to be taken into account when building optimal TF-map alignments. Second, enzyme restriction sites are single-nucleotide positions on the primary sequence. TFBSs, in contrast, are sequence intervals, and thus have in addition to position an associated length. Because we explicitly prohibit overlap between aligned elements, we cannot directly extrapolate the algorithm of [12]. However, as in their approach, we have also taken advantage of the extreme sparsity of the dynamic programming matrix to implement an efficient algorithm that, in our experience, is comparable in efficiency. There is another important feature characteristic of our approach that, while it does not influence the algorithmic strategy, it is essential to its success. As we have already stressed, we do not label the site, but rather the function of the site. That is, we do not label the TFBSs, but we label the TFs that bind to the sites. This allows for significant functional alignments even in the absence of sequence conservation.

We have estimated the optimal parameters of the algorithm in a small, but well-annotated, set of orthologous human–mouse genes (see Materials and Methods). We used three popular collections of PWMs for TFBSs (JASPAR 1.0 [9]), PROMO 2.0 [33], and TRANSFAC 6.3 [10]) to obtain the TF-maps of the promoter sequences. Results on this dataset indicate that, by dramatically reducing the overwhelming number of spurious predictions of TFBSs produced using these collections, TF-map alignments are able to successfully uncover the few conserved functionally active regulatory domains (see Table 3). Differences can be observed between the performance of the different collections of TFBSs; alignments obtained using JASPAR—and, in particular, using a subset consisting of the 50 top most informative matrices—appear to show the optimal balance between sensitivity and specificity. The dataset that we have used, however, is too small to infer general trends on the comparative behavior of these collections.

**Table 3:**
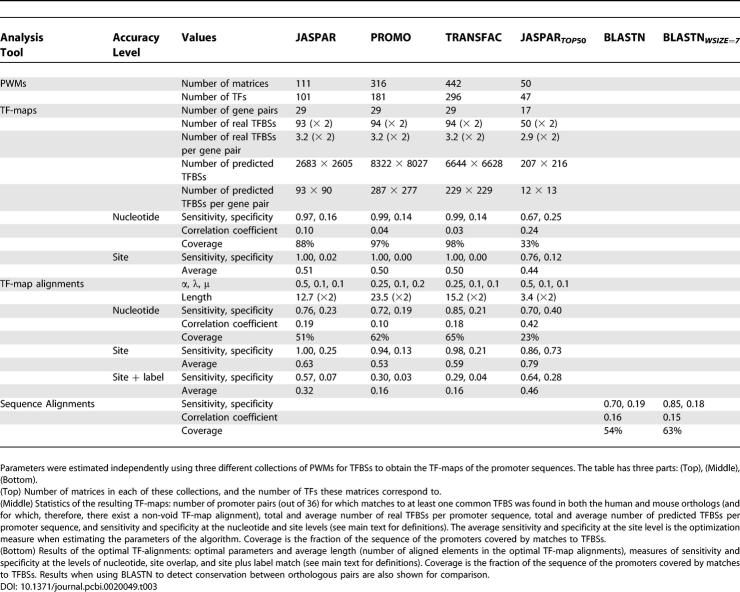
Results of the Estimation of the Optimal Parameters of the TF-map Alignment Algorithm in the HR Set of Orthologous Human–Mouse Promoter Sequences

Interestingly, despite the stronger sequence conservation between protein-coding regions, TF-map alignments score the highest between promoter regions in the training set of orthologous human–mouse genes (see Table 1). This indicates that TF-map alignments are able to pick up regulatory signals that sequence alignments cannot. Results in an independent larger dataset of co-regulated genes from the CISRED database are also in support of this conclusion: we have been able to obtain more significant alignments between the TF-maps than between the nucleotide sequences of the promoters of co-regulated genes. Results in CISRED are certainly not extraordinary. Both sequence and TF-map alignments perform very poorly when detecting relationships between co-regulated genes in CISRED. Only in 97 out of 5,333 gene representatives in CISRED (1.8%) did TF-map alignments score significantly higher for co-regulated than for non co-regulated genes. Using BLASTN, this number was only 11 (0.2%). Finding relationships between the promoters of the genes co-regulated in CISRED is a challenging task, as one can imagine. The CISRED collection of high-confidence co-expressed genes is not derived from overall conservation, or from co-occurrence of motifs, in the sequence of the gene promoters. CISRED co-expression is derived instead from cDNA microarray, SAGE, and other high-throughput gene expression monitoring techniques. CISRED co-expression clusters are thus a mixture of directly and indirectly co-regulated genes, and one would then expect only a few genes within each cluster—maybe in a few subsets—to share functionally equivalent motifs in their promoter sequences. The poor performance of TF-map alignments, however, could also be reflecting the incompleteness of the current collections of TFBSs, and how little we know of the molecular rules governing the expression of human genes.

On the other hand, while building global pairwise alignments may be appropriate to compare promoter sequences of orthologous human–mouse genes, to compare sequences from multiple genes weakly co-regulated—such as those in CISRED—multiple and/or local alignments may be more effective in capturing the functional motifs underlying co-expression. Indeed, from a multiple TF-map alignment of promoters of a set of co-regulated genes, a “transcriptional regulatory superpattern” could be derived capturing those elements conferring expression specificity. Using a local alignment search algorithm, the superpattern could then be used to identify additional genes or transcripts belonging to the same expression class.

Even more appropriate to the analysis of sets of weakly co-expressed genes (that is, including genes both directly and indirectly co-regulated), such as those in the CISRED clusters, would be the extension of the unsupervised pattern recognition techniques usually applied to motif discovery in DNA sequences (in programs such as MEME [34], AlignAce [35], and others, see [6] for a recent comparative evaluation) to motif discovery in TF-maps. This would allow for the identification within a co-expression cluster of different “transcriptional regulatory superpatterns.” These superpatterns, in turn, and the subclusters they induce, could contribute to sort out direct versus indirect co-regulation effects within the cluster. These and other extensions to the TF-map alignments (for instance, those that allow non-colinear arrangements of TFBSs, which have been indeed observed in orthologous genes [36]) are all feasible, and will certainly contribute to the discriminatory power of TF-map comparisons and alignments.

In summary, our results suggest that comparisons of annotations of higher order domains can, on occasion, be more meaningful to characterize the underlying functionality of sequences than direct comparisons at the very primary sequence level. Here we have explored these strategies for the characterization of the promoter regions of co-regulated genes, and we have annotated the primary sequence of them with predictions of TFs. However, we can imagine similar strategies to address many other problems in sequence analysis. One can imagine, for instance, annotating protein sequences with PFAM domains [37], and comparing the resulting annotations to detect distant functional relationships between proteins and protein families. Or annotating genome sequences with the Gene Ontology (GO, [16]) labels of the genes encoded in these sequences, and aligning the GO labels to detect clusters of conserved functions across genomes. In fact, the annotation of the primary sequence with higher order domains to improve alignments has often been explored. For instance, to compare protein secondary structures, see [38], as an example, or to anchor whole genome alignments, see [39,40], or even alignments of promoter regions, see [41]. In all these cases, however, the ultimate goal is to obtain an optimal sequence alignment either between the original primary sequences, or between the 1–1 mappings of the primary sequence into a reduced alphabet (for instance, denoting secondary structure elements). We believe that, as the molecular functionality of the primary sequence becomes better understood, comparisons between higher order annotations, such as those performed here, in which the primary sequence is completely abstracted, may become increasingly relevant.

## Materials and Methods

### Datasets and software availability.

All of the datasets, the promoter sequences, the computational predictions, the TF-map alignments, and the results are freely distributed as flat files at http://genome.imim.es/datasets/meta2005/index.html. An implementation of the enhanced algorithm has been written in C and is publicly available at http://genome.imim.es/software/meta/index.html. A web server that performs the mapping and the meta-alignment of two promoter regions with such an algorithm is accessible at http://genome.imim.es/software/meta/meta.html. The input of the program consists of the two TF-maps to be aligned, each one in a separate file. The files must be in General Feature Format (GFF, http://www.sanger.ac.uk/Software/formats/GFF/GFF_Spec.shtml). Options allow to control the values of *α, λ,* and *μ,* as well as to display the results in plain format or GFF format. The output includes the score and the length of the optimal alignment, and the matches in the two input maps.

### Parameter estimation.

The optimal alignment between two TF-maps is obviously dependant on the *α, λ,* and *μ* parameters. In principle, we want the optimal alignment between the maps derived from promoter sequences of two co-expressed genes to include most of the mapped TFBSs known to be involved in the regulation of the genes (high sensitivity), and few of the mapped TFBSs not known to be involved in such regulation (high specificity). The implicit assumption here is that the TFBSs in the alignment are considered predictions of TFBSs on the underlying promoter sequences. It is also important to stress that two different TFBSs can be aligned if they correspond to the same TF.

The optimal parameter configuration, however, is likely to depend on the particular problem to be addressed: the genes to be compared (orthologous genes from different species or genes co-regulated after an expression microarray experiment, for instance), and the particular protocol to map the TFBSs into the original promoter sequences. Often the optimal configuration of parameters will be specific to the pair of gene promoters to be compared.

With these caveats in mind, because our focus here is on mammalian comparisons, we have estimated the parameters that are globally optimal when aligning a set of well-annotated human–mouse orthologous promoter pairs. The underlying assumption is that these orthologous pairs are regulated in a similar way. We have estimated the optimal parameters separately in three different collections of PWMs for locating TFBSs, and in each case we have chosen the parameters such that the resulting global alignment achieved the maximum average sensitivity and specificity as defined below.

### Datasets.

From several landmark papers in the field [23,24,42–44], we have gathered and manually curated a collection of 278 TFBSs (139 + 139 orthologous sites) that had been experimentally tested in 40 orthologous human and rodent genes. The TSS of each entry in the literature was compared to the RefSeq [45] annotation of the corresponding genome to ensure that we were dealing with the actual proximal promoter. Because most (214 out of 278) of the annotated TFBSs are located in the 200 nucleotides immediately upstream of the TSS, we restricted to this region in our training and evaluation analysis, and considered only those cases for which the same pair of TFBSs had been annotated in this region for both species. This resulted in a collection of 202 sites (101 + 101) from 36 genes, to which we refer here as the HR set. We have estimated the optimal parameters in the HR set for the JASPAR 1.0 [9], PROMO 2.0 [33], and TRANSFAC 6.3 [10] collections. In the three cases, the original frequency coefficients of the matrices have been converted into log-likelihood ratios using the random equiprobability distribution as a background model. The resulting matrices were used to obtain the list of TFBSs matches along the 200 bases upstream of the TSS in each of the 36 pairs of promoter sequences from the HR set. A prediction obtained with a given PWM was accepted if it had a score above 50% (JASPAR), 70% (PROMO), and 55% (TRANSFAC) of the maximum possible score for such PWM. These values correspond in the three cases to the conventional 80% threshold when considering the original frequency matrices.

Those annotated TFBSs not included in the predictions for both orthologous pairs (either because no matrix exists in the collection for such TFBSs or because the match is below the threshold) were discarded. This reduced the effective number of training gene pairs (those with at least one real predicted TFBS for both orthologous pairs) from 36 to 29 for the three collections considered here.

Table 3 shows for each collection the total number of matrices, TFs to which they correspond, the number of genes for which at least one annotated TFBS is predicted on each ortholog after the search, and the number of real and predicted TFBSs (the total and the average per gene pair). As it is possible to see, slightly more than three conserved TFBSs were annotated per orthologous gene pair.

### Accuracy measures.

After the maps were obtained, we aligned them within each orthologous pair using the algorithm described in the previous section with different combinations of parameters. Each parameter was allowed to independently take values between 0.0 and 1.0, in incremental steps of 0.01. In total, thus, one million parameter configurations were evaluated for each collection of PWMs. For each configuration, the resulting optimal alignments on the pairs of orthologous promoters (that is, the predicted TFBSs) were compared with the annotated TFBSs in the promoters.

**Figure 3:**
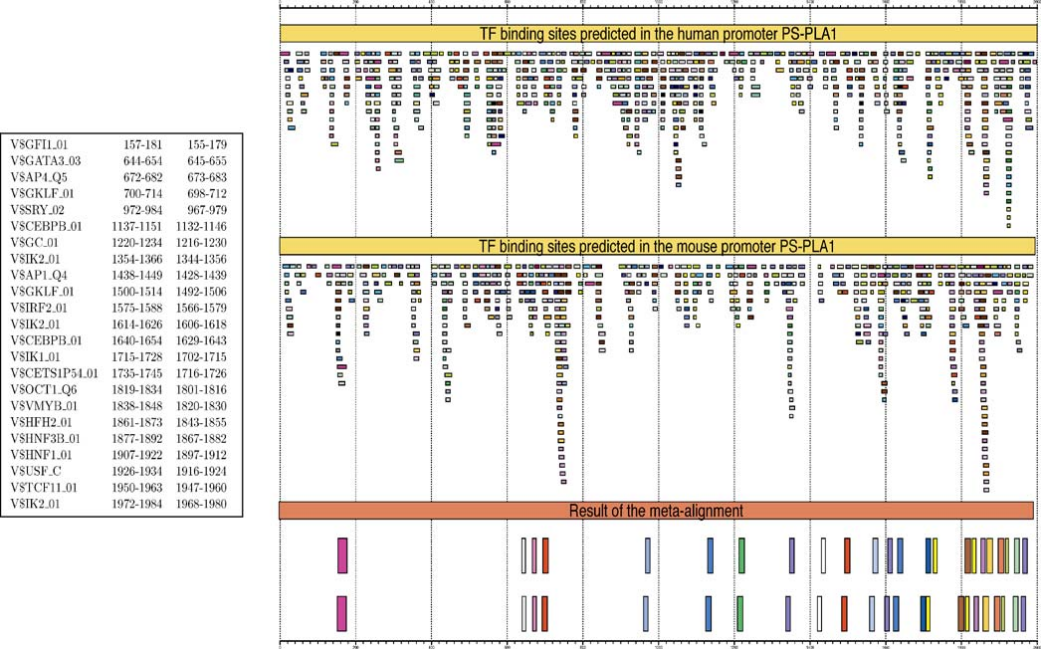
Results of the TF-Alignment of the Human and Mouse Promoters of the *phospholipase A1 member A* Gene Here, the 2,000 nucleotides upstream of the annotated TSS have been considered (with position 1 corresponding to −2,000). The TF-maps on these sequences were obtained using TRANSFAC 6.3 [10]. These maps contained 676 predicted binding sites in human and 595 in mouse (threshold 85%), and they are represented graphically on the top right. Each box represents a different binding site and the color corresponds to the associated TF. The resulting TF-map alignment is also represented graphically at the bottom right. The explicit alignment (the TFs and the coordinates in the human and mouse promoter of the underlying TFBSs) is given on the left. As it is possible to see, while the region proximal to the TSS is not more dense in predicted TFBSs than other regions, most of the aligned elements cluster near the TSS. Indeed, more than half of the elements in the TF-map alignments are within 500 nucleotides of the TSS. The program GFF2PS [48] has been used to obtain the graphical representation of input predictions and final alignment.

Two values were computed to measure the agreement between predicted and annotated TFBSs: sensitivity and specificity. Sensitivity is the number of correctly predicted TFBSs over the number of annotated TFBSs, and specificity is the number of correctly predicted TFBSs over the number of predicted TFBSs. We used here the term specificity as in the gene finding literature. However, the value that we compute here is more generally known as Positive Predictive Value. We considered an annotated TFBS to be correctly predicted when there was a predicted TFBS that overlapped it by at least one nucleotide in both human and mouse sequences, irrespective of whether the TF label associated to the aligned TFBS matched that of the annotated TFBS. This is because TFBSs for different TFs often cluster at the same position when using PWMs (see Figure 3). If a similar cluster occurs in the two sequences to be aligned, our algorithm will inevitably choose to align the pair of TFBSs with the highest sum of match scores (unless the parameter *α* is set to zero, in which case the pair to be aligned will be chosen arbitrarily).

As an optimization measure, we computed the average value of sensitivity and specificity. Table 3 lists the optimal combination of parameters with regard to this measure for each of the three collections of PMWs used here. Table 3 also lists sensitivity, specificity, their average, the average length of the optimal alignments (that is, the number of predicted TFBSs after the alignment), and the fraction of the promoter region covered by the predicted (aligned) TFBSs. In addition, for each optimal configuration we have also computed the same set of accuracy measures under the strict criterion of considering an annotated TFBS to be correctly predicted only when the TF label of the prediction matched that of the overlapped annotation. We also computed sensitivity and specificity at the nucleotide level. At this level, we compute the number of nucleotides in predicted TFBSs that are also in annotated TFBSs. This number over the total number of nucleotides in annotated TFBSs is the sensitivity, and over the total number of nucleotides in predicted TFBSs is the specificity. Finally, as a summary of these two numbers, we compute the correlation coefficient (see [46] for a discussion on these measures). All the accuracy measures were also computed on the initial PWM predictions, prior to the alignments.

### Accuracy results.

As it is possible to see, the main effect of the meta-alignment is the dramatic reduction in the number of predicted TFBSs that typically result after a PWM-based search (see also Figure 3). Taking, for instance, the popular TRANSFAC collection, the average number of TFBSs predicted per promoter in our dataset using this database is about 230. The TF-map alignment reduces this number approximately 15-fold, while the predicted TFBSs still cover essentially all annotated TFBSs. This gain in specificity is not simply due to the selection of an arbitrary set of non-overlapping TFBSs, since as a result of the map alignments the proportion of the promoter region covered by predicted TFBSs drops from 98% to 65%—a number that is more consistent with the estimated occupancy by TFs of the core promoter regions [1].

In this regard, we have compared the map alignments here with direct sequence alignments in their ability to identify TFBSs in the promoter regions of co-regulated genes. We have used NCBI-BLASTN [18] to identify conserved blocks in the promoter region of the orthologous pairs in the HR set. We have searched for local, instead of global alignments because we expect the TFBSs to distribute discretely along the promoter region—resulting in a patch of conserved and non-conserved fragments. In addition, local alignments are insensible to the relative rearrangements in the order of the TFBSs between the promoter sequences compared. This is an advantage over the map alignments, which require colinearity of the TFBSs in the sequences to be compared. Despite this, and the fact that promoter elements are usually embedded within well-conserved sequences in human and mouse orthologous promoters, map alignments are comparable or outperform the BLASTN comparison when identifying TFBSs in them. The correlation coefficient between the sequences covered by the BLASTN alignments and the annotated TFBSs is 0.15, while the same measure when considering the sequences covered by the map alignments is 0.19 for JASPAR, 0.10 for PROMO, and 0.18 for TRANSFAC. Table 3 lists these values, as well as the values of sensitivity and specificity. To obtain these values, BLASTN was run with default parameters, but decreasing the word size to 7 (the minimum accepted value in NCBI-BLASTN). This allows for the detection of shorter and weaker alignments. The performance of BLASTN degrades if we increase the word size. We obtained similar results using the WU-BLASTN version, which allows for shorter word sizes (unpublished data).

The values in Table 3 reflect differences among the three collections of matrices when used in the context of the map alignments. In this context, JASPAR appears to show the better balance between sensitivity and specificity. This can be partially explained because there is less matrix redundancy—which in turn implies less overprediction—in JASPAR than in the other collections. To further minimize overprediction, we have computed the information content of all JASPAR matrices and selected the most informative ones. Let *P* be a PWM where *P*(*x,i*) denotes the probability of observing the nucleotide *x* in the position *i* of a motif of length *n*. The amount of information *R* of the matrix *P* is defined as [47]:

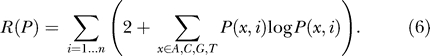
When using the collection of the 50 JASPAR matrices with the highest *R* value (which we refer to as JASPAR*_TOP_*_50_) to obtain the TF-maps, detection of TFBSs through map alignments improves over the entire set of JASPAR matrices: while there is some loss of sensitivity, there is a larger gain in specificity (see Table 3). We have used JASPAR*_TOP_*_50_ in the experiments presented in the Results section.

Finally, we have also performed a complementary test to measure the specificity of the TF-map alignments. As a negative control, we have shuffled the orthologous pairing in the HR set to construct a pool of unrelated human–mouse gene pairs. Then, the corresponding TF-map alignments between these non-orthologous paired promoters were obtained using the parameters previously optimized. For the three collections of matrices, the TF-map alignments between pairs of unrelated promoters were significantly shorter with an average score about 50% smaller than TF-map alignments between “bona fide” orthologous promoters. For instance, the average length of the TF-map alignments between orthologous promoters when using the JASPAR collection was 12.7 TFBSs, with an average score of 55.2. In contrast, the length of the TF-map alignments between non-related promoters was 8.36 TFBSs, with an average score of 20.67. The sites in the alignments involving non-orthologous gene promoters may hypothetically correspond to general regulatory elements present in most core promoters. An alternative, more probable, hypothesis is that they reflect the poor specificity of most PWMs representing TFBSs. Indeed, when we perform the same test using the more informative JASPAR*_TOP_*_50_ collection, no TF-map alignments can be obtained between any pair of the non-related promoters.

## Supporting Information

Dataset S1The 97 Gene Groups in CISRED with More Significant TF-Map AlignmentsThis file lists the 97 genes for which the score of the *coreg*(*x*) alignments was significantly higher than that of 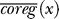 alignments at a significance level of *p* = 0.01. Each line contains the following fields: (1) the ENSEMBL gene identifier; (2) the number of CISRED genes co-regulated with this gene; (3) the average score of the *coreg*(*x*) TF-map alignments; (4) the number of CISRED genes that are not co-regulated with this gene; (5) the average score of the 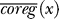 TF-map alignments; (6) the ratio between the average score of the *coreg*(*x*) alignments and the average score of the 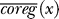 alignments; (7) the significance level *p* according to the Wilcoxon test.
(6 KB TXT)

Figure S1 Number of Accessions to the Matrix *S* during the TF-Map Alignment Training Red, the number of accessions to the matrix *S* by the naive algorithm for each one of the 40 promoter pairs in the HR set.
Orange, the number of accessions of the enhanced algorithm, without sorting the list *L*.
Green, the number of accessions of the enhanced algorithm, sorting the list *L*.
(8 KB EPS)

Figure S2 Distribution of the *coreg*(*x*) TF-Map Alignment Scores The scores of the optimal *coreg*(*x*) TF-maps alignments follow, as optimal sequence alignments, a Gumbel or extreme-value distribution.
(12 KB EPS)

Figure S3Distribution of the 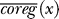 TF-Map Alignment Scores
The scores of the optimal 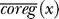 TF-maps alignments follow, as optimal sequence alignments, a Gumbel or extreme-value distribution.
(12 KB EPS)

### Accession Numbers

GenBank (http://www.ncbi.nlm.nih.gov/Genbank) accession numbers are: for ACTA1 (human—AF182035), (mouse—M12347); for PLA1A (human—NM_015900) (RefSeq), (mouse— NM_134102) (RefSeq).

The Ensembl (http://ensembl.org) accession number for TTR is (human—ENSG00000118271).

## Abbreviations

PWMs: position weight matrices
TF: transcription factors
TF-maps: transcription factor maps
TFBSs: TF binding sites
TSS: transcription start site
